# Identification of a genomic region containing genes involved in resistance to four pathotypes of *Plasmodiophora brassicae* in *Brassica rapa* turnip ECD02

**DOI:** 10.1002/tpg2.20245

**Published:** 2022-08-16

**Authors:** Mizanur Rahaman, Stephen E. Strelkov, Hao Hu, Bruce D. Gossen, Fengqun Yu

**Affiliations:** ^1^ Saskatoon Research and Development Centre Agriculture and Agri‐Food Canada Saskatoon SK S7N 0X2 Canada; ^2^ Dep. of Agricultural, Food and Nutritional Science Univ. of Alberta Edmonton AB T6G 2P5 Canada

## Abstract

Clubroot, caused by *Plasmodiophora brassicae*, is an important disease of brassica crops worldwide. Vegetable turnip (*Brassica rapa* L.) have proven to be a source of clubroot resistance genes effective against many pathotypes of *P. brassicae*. The F_1_ progeny from the cross *B. rapa* canola ACDC (susceptible, S) × *B. rapa* turnip ECD02 (resistant, R) were backcrossed with ACDC, then self‐pollinated to produce BC_1_S_1_ lines. All the F_1_ plants were resistant to four pathotypes (3A, 3D, 3H, and 5X) of *P. brassicae*. Segregation for R and S in BC_1_ to each pathotype was 1:1 and resistance reactions were highly correlated. From whole genome sequencing, 192.1 M sequences with 96% template coverage from ECD02, and 478.9 M sequences with 92% coverage from ACDC, were aligned with the reference genome of *B. rapa*. Genotyping‐by‐sequencing was performed on the BC_1_ population. The number of aligned short reads per plant in the BC_1_ ranged from 1.4 to 8.5 M sequences with 4–8% template coverage. We obtained 1,344 high‐quality single‐nucleotide polymorphism (SNP) loci with a mean missing rate at 0.27% and distributed them on 10 chromosomes. A single co‐localized quantitative trait loci (QTL), designated as *Rcr9^ECD02^* on chromosome A08, conferred resistance to the four pathotypes. The QTL explained 68.9–74.4% of phenotypic variation with the logarithm of the odds values of 24.3 to 31.1. Bulked segregant analysis was performed, and 14 SNP markers linked to the gene were developed using the Kompetitive Allele Specific PCR. *Rcr9^ECD02^* was mapped into an interval of 2.2 cM, flanked by CF_A08_10664692 and CF_A08_12230973, which spanned 1.51 Mb on the chromosome and included 219 *B. rapa* genes. Four of these genes (*BraA08g012910.3C*, *BraA08g012920.3C*, *BraA08g013130.3C*, and *BraA08g013630.3C*) encoded disease resistance proteins.

## Core Ideas

- A single quantitative trait loci for clubroot resistance, *Rcr9^ECD02^*, was identified on A08 chromosome of *B. rapa* genotype ECD02.
- The *Rcr9^ECD02^* conferred 69–75% resistance to *P. brassicae* pathotypes 3A, 3D, 3H, and 5X.
- Fourteen robust single‐nucleotide polymorphism markers linked to *Rcr9^ECD02^* were developed for marker‐assisted selection.
- The identified gene was highly effective against the main pathotypes of *P. brassicae* currently identified on canola.
- *Rcr9^ECD02^* could reduce the impact of *P. brassicae* on canola production in this important region.

**Abbreviations**
AAFC: Agriculture and Agri‐Food Canada
Add: additive
BLAST: Basic Local Alignment Search Tool
CCD: Canadian clubroot differential
CDS: coding sequence
CF: Chiifu
CI: confidence interval
CR: clubroot resistant
DH: doubled haploid
DSI: disease severity index
ECD: European clubroot differential
GBS: genotyping‐by‐sequencing
KASP: Kompetitive Allele Specific PCR
LOD: logarithm of the odds
QTL: quantitative trait loci
SNP: single‐nucleotide polymorphism
SRDC: Saskatoon Research and Development Centre

## 1 INTRODUCTION

*Brassica rapa* (genome designated as AA, *n* = 10) is one of the diploid progenitors of *B. napus* (Nagaharu, 1935), a species that plays a significant role in vegetable oil production in Canada and worldwide. The A genome also occurs in *B. napus* (AACC, 2*n* = 38) and *B. juncea* (AABB, 2*n* = 36) (Nagaharu, 1935), contributing to the genetic diversity of these amphidiploid species (Chen et al., 2013; Qian et al., 2006). *B. rapa* is thought to have originated in the Mediterranean region and gradually spread to Scandinavia, Central Europe, Japan, China, and India (Gomez‐Campo & Prakash, 1999; Prakash & Hinata, 1980).

Clubroot disease caused by the obligate parasite *Plasmodiophora brassicae* Woronin is a threat to canola production in Canada (Strelkov & Hwang, 2014), where it can cause a substantial yield loss (30–100%) (Strelkov et al., 2007a; Tewari et al., 2005). Clubroot was first identified on canola in Alberta in 2003 (Tewari et al., 2005) and is spreading rapidly across the Canadian Prairies (Gossen et al., 2015; Hollman et al., 2021). As a result, clubroot represents an important potential constraint to canola production in Canada and could have a significant economic impact, as this crop is valued at $15.4 billion (CDN) annually (Rempel et al., 2014). Clubroot also occurs in many other regions of the world, such as Australia, southeast Asia, northern and central Europe, the United States, and Latin America (Botero et al., 2019; Chittem et al., 2014; Diederichsen et al., 2014; Donald, 2005; Howard et al., 2010; Tanaka et al., 2001; Wallenhammar, 1996; Yang et al., 2012).

The release of the first clubroot resistant (CR) canola cultivar ‘45H29’(Pioneer Hi‐Bred, ON, Canada) in Canada in 2009 (Strelkov et al., 2018) was quickly followed by other CR cultivars with a similar resistance profile (Deora et al., 2012). In 2013, the breakdown of the existing CR resistance by a new and virulent pathotype, named pathotype 5X, was confirmed in Canada near Edmonton, Alberta (Strelkov et al., 2018). The identification of additional new virulent pathotypes that could not be characterized using the existing differential systems led to the development of a new differential system known as the Canadian clubroot differential (CCD) set (Strelkov et al., 2018). Using the CCD, a number of new virulent pathotypes of *P. brassicae* have been identified in Canada (Hollman et al., 2021; Strelkov et al., 2018). Many of these pathotypes, including 3A, 3D, and 5X, are highly virulent on the initial group of CR canola cultivars (Strelkov et al., 2016; Strelkov et al., 2018). Researchers across the region are working to develop durable CR management strategies to reduce the losses caused by these new pathotypes.

Soil amendments (Campbell & Grethead, 1989; Donald & Porter, 2014; Fox et al., 2021; Wellman, 1930), cultural practices (Ahmed et al., 2011; Dixon, 1991; Donald & Porter, 2014; McDonald et al., 2004; Murakami et al., 2002; Tremblay et al., 2005;), fungicides and fumigants (Donald & Porter, 2014; Hwang et al., 2011; Naiki & Dixon, 1987; Peng et al., 2014;) as well as biological control methods (Peng et al., 2014) have been all assessed for their efficacy against clubroot. However, the planting of CR cultivars remains the most effective, inexpensive, and environmentally friendly approach (Peng et al., 2014; Rahman et al., 2014; Strelkov et al., 2016), which is why it has become the most commonly used clubroot management strategy in Canada.

After infection of the root hairs by the pathogen, resistant host plants initially show basal resistance (Dekhuijzen, 1979; Fuchs & Sacristan, 1996; Gravot et al., 2011; Kobelt et al., 2000; Lahlali et al., 2017) to all pathotypes, followed by race or pathotype‐specific resistance (*R‐ gene*‐mediated resistance) (Crute et al., 1980; Rahman et al., 2014; Peng et al., 2014). More than 20 CR loci have been identified and mapped (Sakamoto et al., 2008; Diederichsen et al., 2009; Piao et al., 2009; Hatakeyama et al., 2017; Yu et al., 2017; Karim et al., 2020) in the A genome against pathotypes of *P. brassicae*. Most of the CR loci have been identified on chromosome A03, including *CRa* (Matsumoto et al., 1998), *CRb* (Piao et al., 2004), *CRd* (Pang et al., 2018) *CRk* (Sakamoto et al., 2008), *Crr3* (Hirai et al., 2004; Saito et al., 2006), *PbBa3.1* to *PbBa3.2* (Chen et al., 2013), *Rcr1* (Chu et al., 2014), *Rcr2* (Huang et al., 2017), *Rcr4* (Yu et al., 2017), and *Rcr5* (Huang et al., 2019). Others have been mapped to chromosome A08, including *Crr1* (Suwabe et al., 2003), *CRs* (Laila et al., 2019), *Rcr9* (Yu et al., 2017), *Rcr3* (Karim et al., 2020) and *Rcr9^wa^* (Karim et al., 2020), chromosome A02, including *CRc* (Sakamoto et al., 2008) and *Rcr8* (Yu et al., 2017), and chromosomes A01 (*Crr2*; Suwabe et al., 2006) and A06 (*Crr4*; Suwabe et al., 2006).

Vegetable turnips (*B. rapa* subsp. *rapifera*) have proven to be one of the best sources for identifying *CR* genes effective against many pathotypes of *P. brassicae* in Canada and across the world. Several turnip lines have been included in the European clubroot differential (ECD) set for differentiating pathotypes of *P. brassicae* (Buczacki et al., 1975; Jones et al., 1982). One of these turnip lines, ECD02, is also included in the CCD set. ECD02 was resistant to all 36 pathotypes identified in Canada to date (Strelkov et al., 2018; Askarian et al., 2021; Hollman et al., 2021). Therefore, this genotype represents a very important source for developing canola cultivars with resistance to clubroot in Canada.

The *CRa* resistance gene, originating from ECD02 (reviewed by Piao et al., 2009) and providing resistance to Japanese isolates of *P. brassicae*, was identified (Matsumoto et al., 1998) in Chinese cabbage (*B. rapa* subsp. *pekinensis*) and located on chromosome A03. Molecular markers linked to *CRa* were developed (Matsumoto et al., 2005), and the gene, which encodes a toll‐interleukin‐1 receptor, nucleotide binding site and leucine‐rich repeat (TIR‐NBS‐LRR, TNL) protein (Ueno et al., 2012), was isolated from the Chinese cabbage donor. A study of resistance to Canadian pathotypes 3H, 5X, and 5G confirmed the presence of *CRa* and *Crr1* in the ECD02 population (Fredua‐Agyeman et al., 2020). Two genes for resistance to a field isolate (likely pathotype 3H) of *P. brassicae*, *BraA.CR.a* (A03), and *BraA.CR.b* (A08), were also identified in ECD02 (Hirani et al., 2018). However, no research on the resistance in ECD02 to important new pathotypes has been reported.

The current study was undertaken to identify and map *CR* genes in *B. rapa* with efficacy against the most prevalent pathotypes (3A, 3D, 3H, and 5X) of *P. brassicae* on the Prairies, using genotyping‐by‐sequencing (GBS), quantitative trait loci (QTL) analysis, and conventional linkage mapping approaches. Potential candidate gene(s) in the target region were identified and bulked segregant analysis performed to identify single‐nucleotide polymorphism (SNP) markers and develop markers tightly linked to the gene(s). The mapping of gene(s) effective against these important new pathotypes will assist breeders in developing brassica crop cultivars for effective clubroot management.

## 2 MATERIALS AND METHODS

### 2.1 Parental lines and development of BC_1_/BC_1_S_1_ population

Seed of ECD02, a turnip (*B. rapa*) cultivar, was provided by Dr. G. R. Dixon (The University of Warwick, Wellesbourne, Warwick, UK). ECD02 was identified as resistant to pathotypes 3A, 3D, and 5X from Alberta, as well as 3H, which is the name for the original pathotype 3 in the nomenclature of the CCD (Strelkov et al., 2018). ECD02 was crossed with a highly susceptible *B. rapa* line, ACDC, which was a self‐compatible, doubled haploid (DH) line developed by Dr. K. Falk at the Saskatoon Research and Development Centre (SRDC), Agriculture and Agri‐Food Canada (AAFC). ECD02 is a winter‐type vegetable, so vernalization was needed to induce flowering. After 8 wk of vernalization (4 °C;16 hr light/8 hr dark), the plants were brought back to the greenhouse (21 °C/18 °C) to flower. The plants were covered with a transparent plastic pollination bag at the bud initiation stage to exclude pollen from other plants. Line ACDC was also planted in the greenhouse and allowed to flower, with each flower cluster covered with a pollination bag to prevent cross‐pollination. The F_1_ population was produced by crossing the lines ACDC × ECD02 where the line ECD02 served as a pollen donor. The resulting F_1_ plants were backcrossed with line ACDC to develop a BC_1_ population using the same protocol as for the initial cross. The F_1_ plants were vernalized for 8 weeks as described previously. Some BC_1_ plants did not produce buds until 6 ws after planting, so those plants were vernalized at 4 °C and16 hr light/8 hr dark for 8 wk. All the BC_1_ plants were selfed by covering each plant with a plastic pollination bag to produce a BC_1_S_1_ population. The BC_1_ were self‐incompatible, so a 3% NaCl solution was sprayed to each of the BC_1_ plants to overcome the self‐incompatibility barrier (Yu et al., 2017). This provided a good amount of seed from each plant for phenotyping against the selected pathotypes of *P. brassicae*. Ninety‐three BC_1_S_1_ lines were developed.

**Core Ideas**

- A single quantitative trait loci for clubroot resistance, *Rcr9^ECD02^*, was identified on A08 chromosome of *B. rapa* genotype ECD02.
- The *Rcr9^ECD02^* conferred 69–75% resistance to *P. brassicae* pathotypes 3A, 3D, 3H, and 5X.
- Fourteen robust single‐nucleotide polymorphism markers linked to *Rcr9^ECD02^* were developed for marker‐assisted selection.
- The identified gene was highly effective against the main pathotypes of *P. brassicae* currently identified on canola.
- *Rcr9^ECD02^* could reduce the impact of *P. brassicae* on canola production in this important region.

### 2.2 Evaluating reaction to pathotypes

Seed from the 93 BC_1_S_1_ lines and the parental lines (ECD02 and ACDC) was used to assess the reaction to four important *P. brassicae* pathotypes from Canada, 3A, 3D, 3H, and 5X (field isolate F.3‐14 for pathotype 3A; field isolate F.1‐14 for 3D; field isolate P.41‐14 for 3H; and field isolate LG02 for 5X).

A universally susceptible line, DH16516, originating from the *B. napus* cultivar ‘Topas’, which was provided by Dr. G. Séguin‐Swartz at AAFC, SRDC was included as a susceptible check. A DH line, NRC11‐24, developed by Nutrien Ag Solutions and with known resistance to the pathotype 3H, but susceptible to 3A, 3D, and 5X, was used as a second susceptible check to verify the pathogenicity of pathotypes 3A, 3D, and 5X, and as a resistant check for 3H. Two DH lines (AAFC‐Y12 and AAFC‐Y68) developed at AAFC, which carry the *CR* genes *Rcr9^ECD01^* and *Rcr10^ECD01^* and are highly resistant to pathotypes 3A, 3D, 3H and 5X (Yu et al., 2021), were included as resistant controls. Three CR genes, *Rcr4* for resistance to 3H, and *Rcr8* and *Rcr9* for resistance to 5X, were identified in *B. rapa* breeding line T19, which originated from the German turnip ‘Pluto’ (Yu et al., 2017). Sixteen BC_1_S_1_ lines from ACDC x T19 carrying the genes individually or in combinations (Table S1) were also included as controls.

Twelve seedlings of each line, including the parents, were inoculated individually with each of the four pathotypes under controlled conditions in a growth chamber at AAFC, SRDC. The inoculum suspension was prepared following a standard protocol (Strelkov et al., 2016) modified by Karim et al. (2020). Briefly, frozen galls were cut into small pieces and soaked in water in a glass beaker for 20–30 min, then homogenized in a blender for 2–3 min and strained through two layers of nylon cloth. The spore concentration was estimated using a hemocytometer, adjusted to 1 × 10^7^ resting spores mL^−1^, and stored at −20 °C until required.

Seedlings of each line were grown in 10 cm × 10 cm plastic pots filled with a commercial soil‐less mix (Sunshine Mix 3, TerraLink Horticulture), packed and set in a plastic tray to catch and contain any runoff, with 12 seedlings per pot and 32 pots per tray. The pots were thoroughly soaked with water, allowed to drain, and excess water was removed. After seeding, each tray was covered with a transparent plastic dome in a growth chamber set at 21 °C ± 2 °C under 16 hr light/8 hr dark for 1 wk. At 1 wk after planting, the seedlings were inoculated with 15 ml of spore solution per pot. Immediately after inoculation, the trays were covered again and moved back to the growth chamber. Watering of the seedlings and trimming of leaves were carried out as needed. At 5 wk after inoculation, the plants were uprooted, washed under tap water, and scored using the 0–3 rating scale described by Kuginuki et al. (1999), where 0 = no club symptoms on roots, 1 = a few small clubs, 2 = moderate clubbing, and 3 = severe clubbing on the whole roots (Figure S1).

Clubroot severity was calculated for each line as a disease severity index (DSI) following Horiuch and Hori (1980) as modified by Strelkov et al. (2006) based on the following formula:

$$\mathrm{DSI}=\frac{\sum \left(n\times 0+n\times 1+n\times 2+n\times 3\right)}{N\times 3}\times 100$$

where DSI is the overall severity on a line; *n* is the number of plants in each class; *N* is the total number of plants assessed; and 0, 1, 2, and 3 are the disease severity classes.

The lines from the BC_1_S_1_ population were separated into two categories: resistant (R) or susceptible (S). A line was regarded as R when the DSI < 60% and susceptible when the DSI ≥ 60% (Yu et al., 2017). The segregation ratio of R and S was calculated, and goodness of fit was tested with a χ^2^ test using Microsoft Excel software. In addition, the correlation coefficients among DSI values of the tested lines were calculated and the significance of the correlation was tested with a t‐test (Iversen & Gergen, 1997) using Microsoft Excel. The study was repeated, with similar results obtained in both repetitions. The only important difference between the runs was that the DSI values were generally higher in the repetition. For lines with inconsistent results, a low DSI was considered to be less reliable because it might result from disease escape or suboptimum conditions for infection. Therefore, the results of the second repetition were used to identify and map QTLs from the population

### 2.3 Genomic DNA extraction, DNA sequencing, and short read assembly

Nucleic DNA was extracted from young leaf tissues of the 93 BC_1_ plants and the parental lines using QIAGEN DNeasy kits following the manufacturer's directions. The quality of the extracted DNA was evaluated with a Nanodrop2000 (Thermo Scientific) spectrophotometer and by gel electrophoresis, and the amount of DNA was adjusted to provide high‐quality sequences from each sample. Genotyping‐by‐sequencing was performed on an Illumina HiSeq 2000SE using paired‐end lane sequencing at BGI. Whole‐genome sequencing of the parent lines ECD02 and ACDC was performed at the Plant Biotechnology Centre (Saskatoon, SK, Canada). The short reads obtained from the sequencing were assembled and aligned using SeqMan NGen 16 (DNASTAR, Madison, WI) against the *B. rapa* reference genome v3.0 (‘Chiifu’ [CF]) downloaded from http://brassicadb.org/brad/downloadOverview.php (Zhang et al., 2018).

### 2.4 SNP filtering, linkage map construction, and QTL detection

The assembled and aligned sequences were analyzed using ArrayStar 16 (DNASTAR) to identify SNP variants in the sequences when compared with the *B. rapa* genome v3.0. The sequences of the 93 BC_1_ plants were filtered to leave only high‐quality SNPs; the variants were reduced by at least 50% based on the SNP criteria of depth coverage >5, quality score (Q) > 30, and the SNP percentage >10 on the ArrayStar platform. The remaining SNPs were further filtered on JoinMap 4.1 to select tightly linked SNPs, and a linkage map was drawn using Mapchart 2.1 (Voorrips, 2002) using only SNPs with <20 cM distance between the adjacent markers. The GBS‐SNP sites were named based on the A‐genome chromosome (A01 to A10) and position on the reference chromosome sequence on the reference genome (CF); SNP loci from the R parent (ECD02) were scored as H and those from the susceptible parent (ACDC) as A. The filtered SNPs were used in association with the phenotypic data to map QTLs associated with the clubroot‐resistance trait using IciMapping (Inclusive Composite Interval Mapping) method (Meng et al., 2015). IciMapping was run at 1,000‐permutations (known as bootstrapping) with a type I error rate of 0.01 for QTL declaration. The QTLs identified in this analysis were authenticated based on a maximum logarithm of the odds (LOD) value with the phenotypic variation explained, additive (Add) effect of the QTL, and confidence interval (CI) on the trait of interest.

### 2.5 Bulked segregant analysis and Kompetitive Allele Specific PCR (KASP)

Quantitave trait loci analysis identified a strong QTL for resistance to the pathotypes on chromosome A08. The phenotype (R or S) of each BC_1_ plant was determined, based on the mean DSI against each of the four pathotypes (3A, 3D, 3H, and 5X) assessed on the BC_1_S_1_ lines. As noted above, a line was regarded as R when the DSI < 60% and susceptible when the DSI ≥ 60%. An R line was scored as H and an S line as A. Linkage analysis was performed with the SNP markers and phenotypes using JoinMap 4.1 (Van Ooijen & Voorrips, 2001).

To identify SNP sites tightly linked to the QTL and develop robust SNP markers for use in marker‐assisted selection, 43 resistant plants were combined to form an R bulk and 50 susceptible plants to form an S bulk, and the GBS short reads from each bulk were then aligned with the *B. rapa* CF reference genome using SeqMan NGen 16. Selected SNPs identified in the target region were confirmed using the KASP method (http://www.lgcgroup.com/) following the manufacturer's instructions. Polymerase chain reactions were performed in a StepOne Plus Real Time PCR System (Applied Biosystem). Linkage analysis with the confirmed SNP markers was performed using KASP analysis and the phenotypes determined based on the mean DSIs of the four pathotypes using JoinMap 4.1 (Van Ooijen & Voorrips, 2001).

### 2.6 Identification of genes encoding disease resistance proteins in the target region

Genes encoding disease resistance proteins were identified using Basic Local Alignment Search Tool (BLAST) 2GO 4.1.9 with a minimum E‐value of 1 × 10^−3^ (Conesa et al., 2005) against the *Arabidopsis thaliana* gene model. This process annotated the function of the genes in the QTL target region using the coding sequence (CDS) of genes of the *B. rapa* CF. Genes encoding disease resistance proteins in the targeted region were examined to identify potential candidate genes for each QTL. To keep the comparison consistent in the CR gene comparisons, all of the previously identified genes identified using other forms of the reference genome were converted to the latest version (v3.0; CF) and compared with the QTL identified in the current study. The most probable Arabidopsis homolog corresponding to each disease resistance gene and the class of disease resistance proteins were determined using the CDS of the disease resistance genes in *B. rapa* by BLAST search at http://www.arabidopsis.org.

## 3 RESULTS

### 3.1 Clubroot disease reaction

**FIGURE 1:**
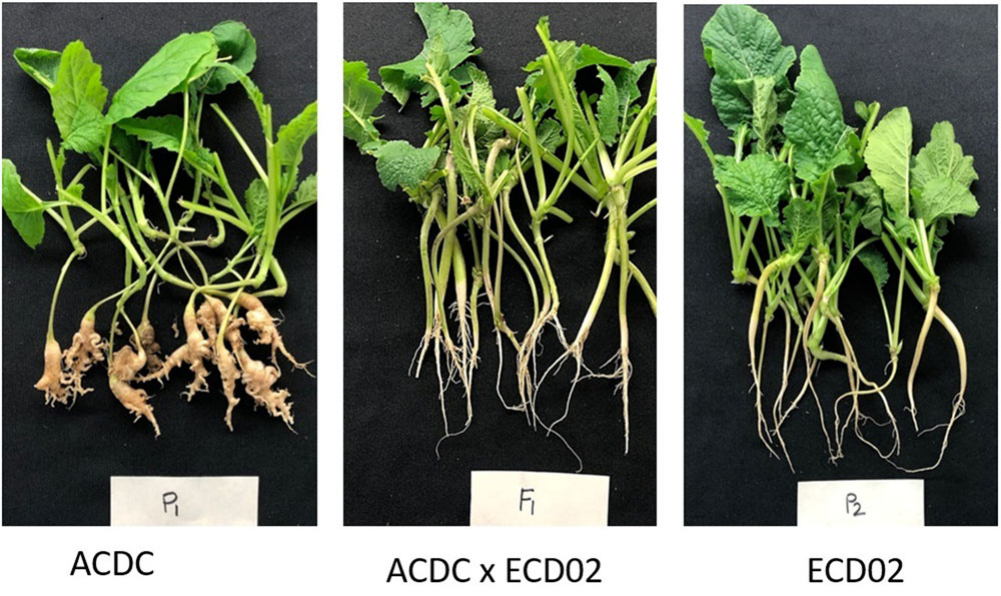
Typical clubroot reaction of the *Brassica rapa* parental lines and their F_1_ progeny inoculated with pathotypes 3A, 3D, 3H, and 5X of *Plasmodiophora brassicae*

**FIGURE 2:**
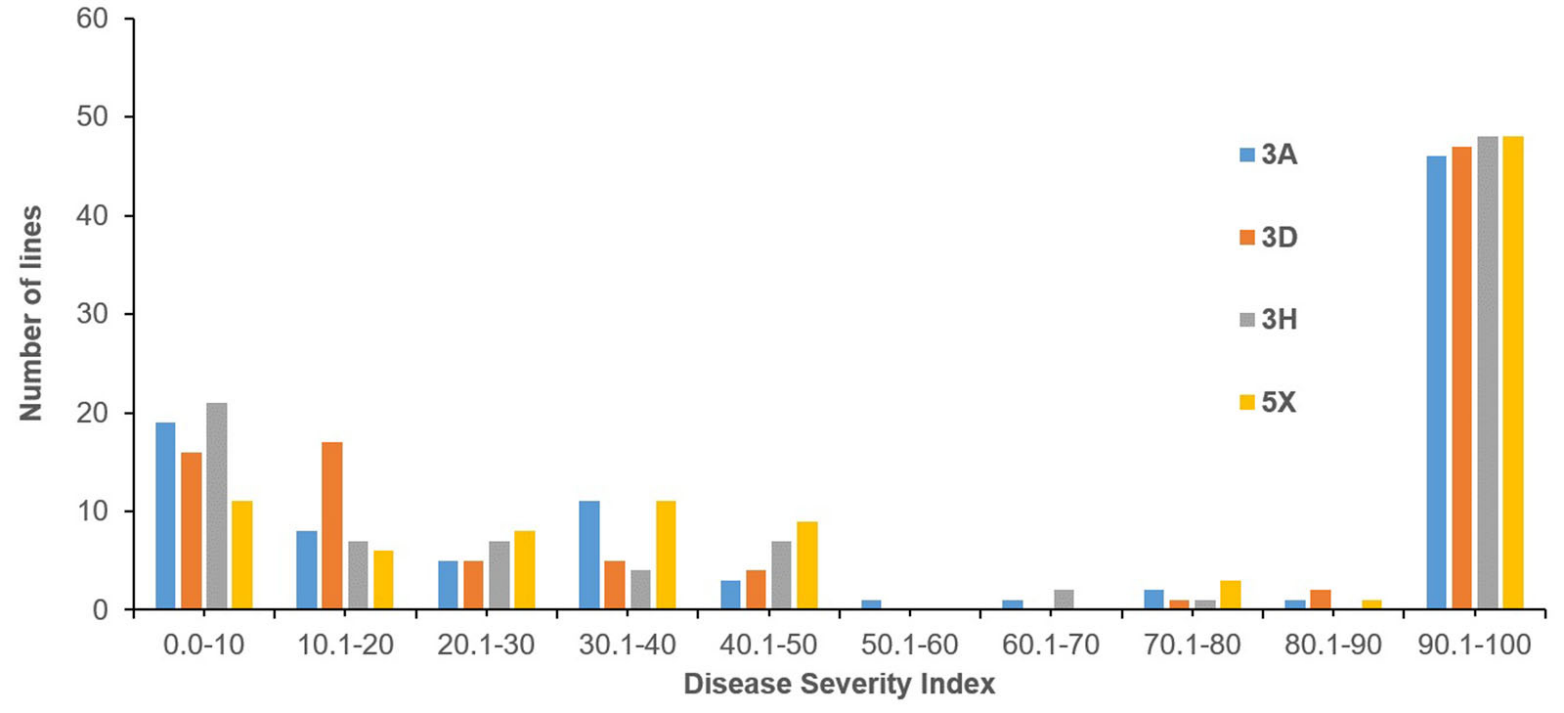
Clubroot disease severity index on 93 lines of a BC_1_ population derived from *Brassica rapa* ECD02 and inoculated with pathotypes 3A, 3D, 3H, and 5X of *Plasmodiophora brassicae*

In the assessment of the reaction to pathotypes 3A, 3D, 3H, and 5X, all of the plants of the R parent ECD02 were resistant (0% DSI) to all four pathotypes, whereas all plants of the S parent ACDC were highly susceptible (100% DSI). All of the F_1_ plants were resistant to all four pathotypes with 0% DSIs (Table 1, Figure 1). The BC_1_S_1_ population showed a range of reactions (Figure 2). Lines with DSI < 60% were regarded as R and those with DSI ≥ 60% as S (Yu et al., 2017). Based on these criteria, the BC_1_ derived from ACDC × (ACDC × ECD02) had a 1:1 (R:S) segregation ratio against each pathotype, based on the DSIs of their BC_1_S_1_ progenies (Table 1). The 1:1 ratio in BC_1_ and resistance reaction of the F_1_ plants indicated that a single dominant gene derived from ECD02 controlled the resistance to each pathotype.

**TABLE 1:** Number of plants in the Brassica rapa parental lines, F_1_, BC_1_/BC_1_S_1_ progenies, susceptible and resistant checks that were resistant (R) or susceptible (S) to pathotypes 3A, 3D, 3H, and 5X of Plasmodiophora brassicae

Materials	3A		3D		3H		5X	
	R	S	R	S	R	S	R	S
ECD02	12	0	12	0	12	0	12	0
ACDC	0	12	0	12	0	12	0	12
F1 (ACDC × ECD02)	12	0	12	0	12	0	12	0
BC_1_/BC_1_S_1_	43	50	43	50	42	51	41	52
χ^2^	0.53		0.53		0.87		1.30	
*P* value	0.47		0.47		0.35		0.25	

*Note*. The phenotype (R or S) of each BC_1_ plant was determined based on the disease severity index (DSI)of the BC_1_S_1_ line derived from it. A BC_1_S_1_ line was regarded as R when the DSI was < 60% and S when the DSI was ≥ 60%.

The DSI values to the pathotypes in the BC_1_S_1_ population derived from ACDC × ECD02 were highly correlated (0.84 ≤ *r* ≤ 0.94) (Table 2). The strong correlation indicated that the resistance to all four pathotypes (3A, 3D, 3H, and 5X) might be controlled by the same or a group of closely linked genes derived from the resistant parent.

**TABLE 2:** Correlation matrix for clubroot disease severity index in 93 BC_1_S_1_ lines derived from 93 BC_1_ plants of ACDC × (ADCD × ECD02) inoculated with four pathotypes (3A, 3D, 3H, and 5X) of Plasmodiophora brassicae under controlled conditions

Pathotypes	3A	3D	3H
3D	0.93**		
3H	0.95**	0.92**	
5X	0.89**	0.91**	0.87**

** Significant at *P* ≤ .01.

As shown in Table S1, all plants of the susceptible control DH16516 were also highly susceptible (100% DSI) to each pathotype. NRC11‐24 were highly susceptible to pathotypes 3A, 3D, and 5X (100% DSI) but resistant to 3H (0% DSI). The *B. napus* breeding lines Y12 and Y68 were highly resistant to all four pathotypes (0% DSI).

The BC_1_S_1_ lines carrying *Rcr4* were resistant to pathotype 3H but susceptible to 5X. On the other hand, lines carry *Rcr8* or *Rcr9* or both *CR* genes were susceptible to 3H, but resistant to 5X (Yu et al., 2017). Sixteen BC_1_S_1_ lines selected from the population described by (Yu et al., 2017) were further tested with pathotypes 3A, 3D, 3H and 5X. For resistance to 3H and 5X, the result was consistent with that reported previously (Yu et al., 2017). However, lines carrying *Rcr4*, *Rcr8*, or *Rcr9* alone and two or three of the genes in combination showed a high level of susceptibility to pathotypes 3A and 3D (>95% DSIs) (Table S1).

### 3.2 Alignment with reference genome

When DNA short reads from whole‐genome sequencing of the parental lines and GBS from the 93 BC_1_ plants were aligned with the *B. rapa* reference genome. The total number of short reads was 214.3 million (M), with 192.1 M sequences assembled and 96% template coverage of the genome for the resistant parent ECD02, and 552.9 M with 478.9 M sequences assembled and 92% template coverage for ACDC (Table S2). A total of 432.9 M GBS short reads in the 93 BC_1_ plants were obtained, ranging from 1.6 to 9.2 M sequences per line. The mean number of reads aligned with the reference genome from each line was 4.0 M (range 1.4–8.5 M, Table S2). The template coverage in each line ranged from 4.0 to 8.1%, with the mean of 5.7% in the population (Figure S2, Table S2).

### 3.3 Identification of SNPs and construction of linkage groups

**FIGURE 3:**
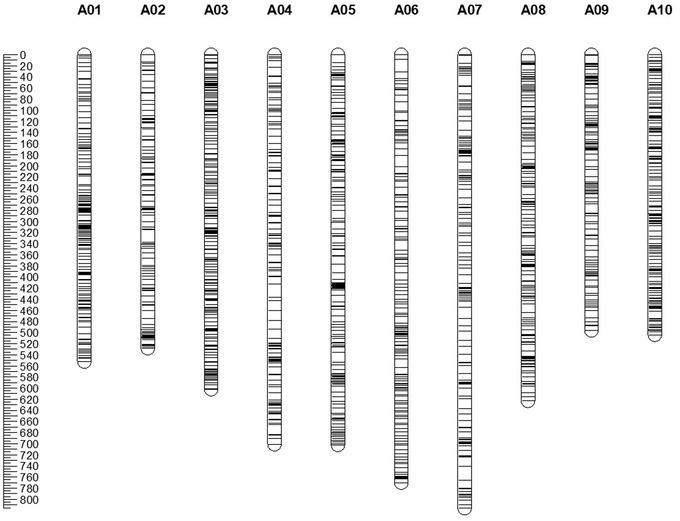
Linkage map derived from single‐nucleotide polymorphisms (SNPs) of the *Brassica rapa* BC_1_ population [ACDC × (ACDC × ECD02)] consisting of 2,496 SNPs

After sequence assembly, the data were assessed to identify variants and to create a SNP table. There were 93,454 SNPs present in at least 50% of the progeny lines. Of these, 16,702 highly polymorphic markers were selected based on their LOD values; markers with >15 LOD values were used to create a linkage map. The 16,702 SNPs were further analyzed, and tightly linked markers were selected, based on an LOD value of 15–20, which selected 2,539 high‐quality markers that were distributed across the 10 chromosomes. The mean number of SNPs on each linkage group was 254, with 306 SNPs on linkage group A10, and 189 SNPs on A07. The mean chromosome length was about 620 cM and ranged from 530 to 820 cM (Figure 3).

Among the 2,539 filtered SNPs, 1,195 duplicate markers were identified and deleted using QTL IciMapping. The remaining 1,344 high‐quality, unique SNPs were distributed into 10 chromosomes of A‐genome, with 105–159 SNPs per chromosome and an average missing rate of 0.27% (Table S3). Each SNP was positioned about 3 to 5 cM apart along each chromosome (Table 3). The estimated length of the chromosomes ranged from 452 to 757 cM with a mean length of 452 cM. These SNPs were used to identify the association between the clubroot reaction of each line and QTL associated with resistance.

**TABLE 3:** Distribution of SNPs on linkage groups constructed from the Brassica rapa BC_1_ population [ACDC × (ACDC × ECD02)]

Linkage group	Number of markers	Total length	Mean distance between two adjacent markers
		cM	
A01	136	515.4	3.79
A02	130	756.6	5.82
A03	159	547.0	3.44
A04	115	630.1	5.48
A05	146	648.2	4.44
A06	141	693.3	4.92
A07	131	743.8	5.68
A08	135	561.8	4.16
A09	105	451.9	4.30
A10	146	475.7	3.26
Total	1,344	6,023.8	–
Maximum	159	756.6	5.82
Minimum	105	451.9	3.26
Mean	134	602.4	4.53

### 3.4 Mapping QTL for resistance to pathotypes 3A, 3D, 3H, and 5X

**FIGURE 4:**
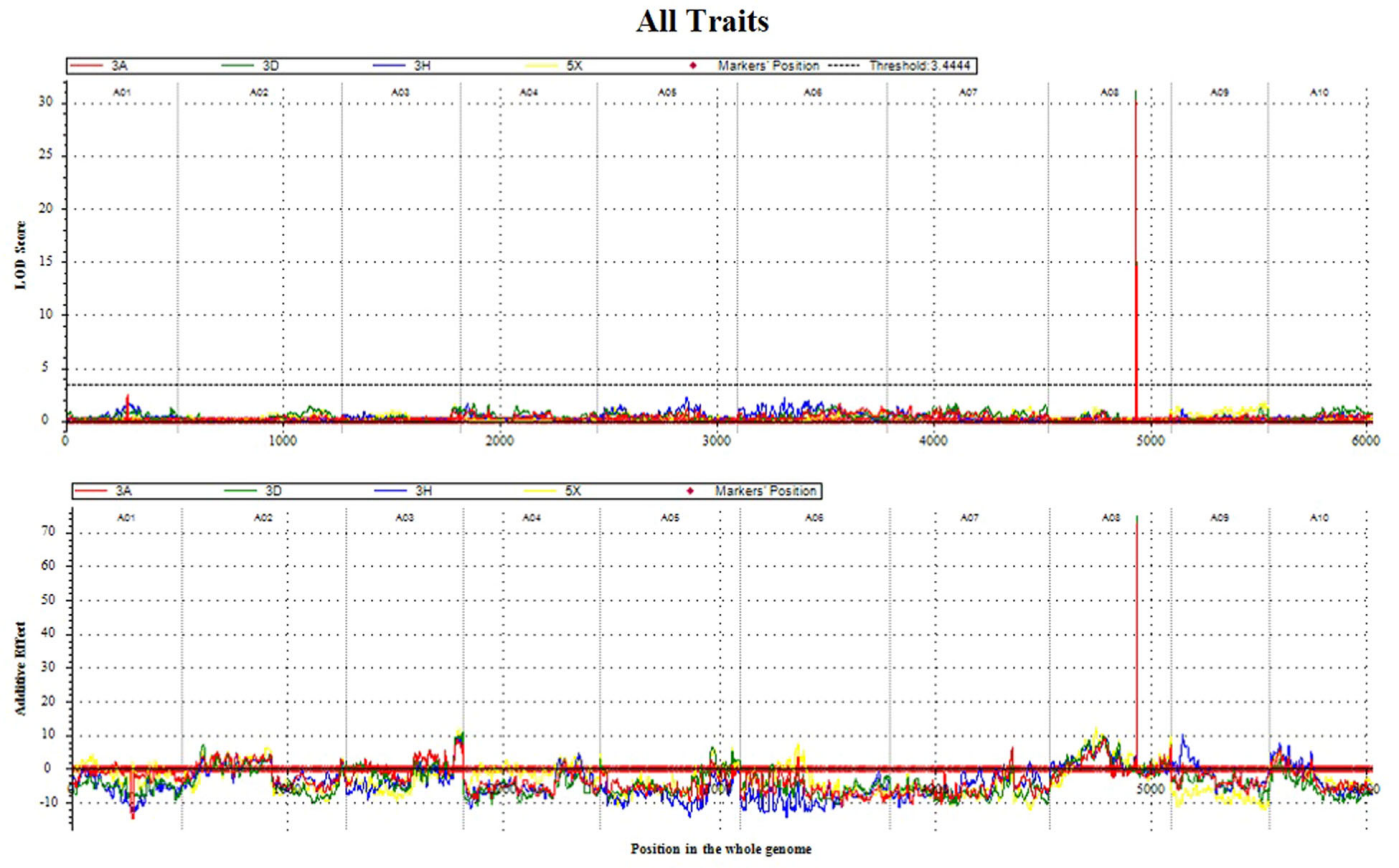
Plots of the logarithm of the odds (LOD) and additive effect of clubroot resistance quantitative trait loci against pathotypes 3A, 3D, 3H, and 5X of *Plasmodiophora brassicae* with a linkage group of BC_1_ population derived from *Brassica rapa* line ECD02

Quantitative trait loci mapping was performed using 1,344 high‐quality, polymorphic SNPs in association with the DSI value for each line to each pathotype. A single co‐localized QTL for resistance to all four pathotypes, designated as *Rcr9^ECD02^*, was mapped on linkage group A08, with a strong peak associated with SNP markers CF_A08_10575267 and CF_A08_11903476. The SNP marker on the left CI was CF_A08_11903476 (399.5 cM) and on the right CI it was CF_A08_10575267 (402.5). These markers were located in the interval of *Rcr9*, a CR gene identified previously (Yu et al., 2017). However, *Rcr9^ECD02^* conferred resistance to all four of the pathotypes assessed, whereas *Rcr9* conferred resistance to 5X only. *Rcr9^ECD02^* was associated with a phenotypic variation explained of 68.9 to 77.4% in response to the individual pathotypes, with corresponding LOD values of 24.3–31.1 (Table 4, Figure 4). The Add values of the QTL to resistance were in the range of 64.2 to 74.7 against the pathotypes (Table 4, Figure 4).

**TABLE 4:** Position on chromosome A08 of quantitative trait loci (QTL) originating from Brassica rapa ECD02, flanking marker designations, phenotypic variation explained (PVE), additive (Add), logarithm of the odds (LOD), and confidence interval (CI) for the QTL (designated Rcr3^ECD02^) for resistance to four pathotypes of Plasmodiophora brassicae (permutations = 1,000)

Pathotype	Position	Left marker	Right marker	LOD	PVE (%)	Add	Left CI	Right CI
3A	401.0	CF_A08_11903476	CF_A08_10575267	30.1	74.3	72.7	399.5	402.5
3D	401.0	CF_A08_11903476	CF_A08_10575267	31.1	77.4	74.7	399.5	402.5
3H	401.0	CF_A08_11903476	CF_A08_10575267	28.0	74.1	74.0	399.5	402.5
5X	401.0	CF_A08_11903476	CF_A08_10575267	24.3	68.9	64.2	399.5	402.5

### 3.5 DNA variants and SNP markers in the target region

To confirm the location of *Rcr9^ECD02^*, the BC_1_–BC_1_S_1_ population was analyzed using conventional linkage mapping. There were 43 R plants and 50 S plants in the BC_1,_ based on the mean DSIs of four pathotypes in the BC_1_S_1_ populations. There were 135 high‐quality SNP markers identified on chromosome A08, but only two SNP markers (CF_A08_10575267 and CF_A08_11903476) were closely associated with *Rcr9^ECD02^*.

To identify more SNP sites in the target region and develop robust SNP markers that could potentially be used for marker‐assisted selection, GBS short reads from the R bulk and the S bulk were aligned with the *B. rapa* CF reference genome. There were 5.5 M short reads and 6.8 M assembled into chromosome A08 with template coverage of 14.9% from the R bulk and 15.3% from the S bulk. Further analysis to identify high‐quality variants uniquely from the R bulk was performed in the target region (10,575,267 to 11,903,476 bp of A08) and an extended region to 12,326,805 bp that included the previously cloned *Crr1* (Suwabe et al., 2003), which was homologous to *BraA08g014480.3C* (12,271,553–12,276,276 bp of A08). There were 44 DNA variants consisting of 41 SNPs and 3 InDels in this region (Table S4).

**FIGURE 5:**
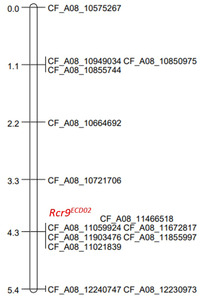
Linkage map of 14 single‐nucleotide polymorphisms (SNPs) from a Kompetitive Allele Specific PCR assay and 2 SNPs identified by quantitative trait loci analysis, illustrating the relative position of gene *Rcr9^ECD02^* chromosome A08

KASP assay was carried out for 22 of the 41 SNPs. Of these 22 SNPs, 14 were polymorphic between the parental lines used for genotyping the 93 BC_1_ plants. A linkage map consisting of the 14 SNPs identified with the KASP assay and 2 SNPs identified by QTL analysis was constructed (Figure 5 and Figure S3). *Rcr9^ECD02^* co‐segregated with CF_A08_11021839, CF_A08_11059924, CF_A08_11466518, CF_A08_11672817, CF_A08_11855997, and CF_A08_11903476, and was flanked by CF_A08_10721706 and CF_A08_12230973, in an interval of 2.2 cM. This interval was upstream of *BraA08g014480.3C* (12,271,553 to 12,276,276 bp of A08), where *Crr1* is located.

### 3.6 Identification of genes encoding disease resistance proteins

*Rcr9^ECD02^* was associated with two SNP markers (CF_A08_10721706 and CF_A08_12230973) in the KASP assay. The region between these two markers, which spanned 1.5 Mb on the chromosome, included 219 *B. rapa* genes identified on the reference genome (Table S5). Among these 219 genes, there were four potential resistance genes: *BraA08g012910.3C*, *BraA08g012920.3C*, *BraA08g013130.3C*, and *BraA08g013630.3C* (Table 5).

**TABLE 5:** A list of genes encoding proteins associated with plant disease resistance obtained from BLAST2GO and Blast searches (https://www.arabidopsis.org/Blast/index.jsp) with a coding sequence in the chromosome region of Arabidopsis homologous to the Rcr9^ECD02^ target region

Gene designation	*B. rapa* gene locn.	Length (base)	Gene description	Homolog in Arabidopsis	R gene class
BraA08g012910.3C	11232393…11235117	2724	disease resistance	AT3G05360	receptor‐like protein 30
BraA08g012920.3C	11248087…11250727	2640	disease resistance	AT3G05360	receptor‐like protein 30
BraA08g013130.3C	11388577…11391460	2883	disease resistance	AT3G05360	receptor‐like protein 30
BraA08g013630.3C	11696216…11696785	569	disease resistance	AT5G11250	a typical TIR‐NBS‐LRR

The first three genes were homologous to the Arabidopsis gene *AT3G05360*, which encodes receptor‐like protein 30. The fourth gene was homologous to *AT5G11250*, encoding an atypical TNL protein, *BraA08g014480.3C*, which was homologous to the previously cloned resistance gene *Crr1*. However, this gene was not in the interval previously reported for *Crr1* (Table 5).

## 4 DISCUSSION

Clubroot, caused by *P. brassicae*, is an important disease of canola in Canada. All of the initial CR canola cultivars released in Canada have proven to be susceptible to many of the new pathotypes of *P. brassicae* that have emerged in recent years, including 3A, 3D, and 5X identified from CR canola (Strelkov et al., 2018). Pathotype 3A is currently predominant among the new pathotypes, followed by 3D (Strelkov et al., 2018; Hollman et al., 2021). Genetic resistance to these pathotypes is urgently needed to protect canola in the region from substantial yield losses caused by these new pathogen strains.

The *B. rapa* line ECD02 was highly resistant to all four of the pathotypes tested in the current study. All of the F_1_ plants were resistant to all four of the pathotypes. Based on our previous observation (Yu et al., 2016; Yu et al., 2017), BC_1_S_1_ lines with DSI < 60% were likely from resistant BC_1_ plants. We therefore classified BC_1_S_1_ lines with DSI < 60% as R and those lines with DSI ≥ 60% as S lines in this study. The BC_1_ plants developed segregated for R:S at a 1:1 ratio. This indicated that resistance was controlled by one dominant gene from the resistant parent ECD02. The DSI values for each line were highly correlated for each of the pathotypes. This indicated that resistance was controlled by a single gene or tightly linked genes. Analysis identified a single co‐localized QTL, *Rcr9^ECD02^*, on chromosome A08 conferring resistance to pathotypes 3A, 3D, 3H, and 5X, which was consistent with the conventional analysis. Both QTL analysis and linkage mapping indicated that *Rcr9^ECD02^* mapped to the 10,575,267 to 12,331,263 base region of chromosome A08 in *B. rapa* CF v3.0. The name *Rcr9^ECD02^* was selected because the gene was mapped into the genetic region of *Rcr9* and was originally derived from *B. rapa* line ECD02.

Clubroot severity in the DH lines in response to the individual pathotypes was highly correlated, which indicated that the same or tightly linked genes likely controlled resistance to these pathotypes. However, the identification of QTL in this study was based on relatively coarse gene mapping, so it could not be determined if resistance to the pathotypes was controlled by a single gene or tightly linked genes. Additional studies are in progress. However, strong resistance to Canadian clubroot pathotypes is generally controlled by single dominant genes such as *Rcr1*–*Rcr9* (Chu et al., 2014; Yu et al., 2016; Huang et al., 2017; Yu et al., 2017; Dakouri et al., 2018; Chang et al., 2019; Huang et al., 2019; and Karim et al., 2020).

Previous studies identified *CRa* on chromosome A03 in ECD02 (Matsumoto et al., 1998; Matsumoto et al., 2005; Ueno et al., 2012; Fredua‐Agyeman et al., 2020). However, *CRa* was not found in the current mapping population. In nature, *B. rapa* is self‐incompatible and requires cross‐pollination. As a result, it is unlikely that ECD02 is a homogenous line. It is therefore possible that the mapping population derived from the donor plant used for this study did not carry *CRa*.

A previous study by our group identified three QTLs/genes in *B. rapa* line T19 that conferred resistance to several pathotypes (Yu et al., 2017). T19 had originated from the German turnip ‘Pluto’. A single co‐localized QTL on chromosome A03, designated *Rcr4*, conferred resistance to pathotypes 2F, 3H, 5I, 6 M, and 8N (Yu et al., 2017) but was not effective against 3A, 3D, or 5X (Table S1). Although *Rcr8* on chromosome A02 and *Rcr9* on A08 also conferred resistance to pathotype 5X (Yu et al., 2017), they could not confer resistance to 3A, 3D or 3H (Table S1). Therefore, *Rcr9^ECD02^* from ECD02 is likely a different gene than the three genes from T19.

Our group had identified *Rcr9* for resistance to pathotype 5X in the *B. rapa* breeding line T19 (Yu et al., 2017). The proposed position of *Rcr9* spanned a large interval (6.48 Mb) of chromosome A08 (Figure S4), including the genome region of *Rcr3*, *Rcr9^wa^* (Karim et al., 2020), and *Rcr9^ECD01^* (Yu et al., 2021). However, the BC_1_S_1_ lines that carried *Rcr9* were resistant to 5X but not to 3A, 3D, or 3H (Table S1). This difference in phenotype indicated that *Rcr9* differed from *Rcr9^ECD02^*. In addition, another resistance gene, designated as *Rcr9^wa^*, has been identified from a differential line in the ECD set. It originated from the turnip ‘Waaslander’ (ECD04), was mapped to the same interval as *Rcr9*, and provided resistance to pathotype 5X (Karim et al., 2020). Based on flanking markers, *Rcr9^wa^* was mapped to the 12.3–12.6 Mb region of chromosome A08 (a smaller interval than *Rcr9*), which is distinct and separate from *Rcr9^ECD02^*. Similarly, another resistance gene that originated from Waaslander and conferred resistance to pathotype 3H, designated *Rcr3*, was mapped to chromosome A08 and was flanked by SNP markers on the 11.3–11.6 Mb region in the *B. rapa* CF reference genome v3.0 (Karim et al., 2020). The position of *Rcr3* was in the interval of *Rcr9^ECD02^*. However, it conferred resistance to pathotype 3H, but not to 5X (Karim et al., 2020). Finally, our group has identified a QTL from ECD01, designated as *Rcr9^ECD01^*, which conferred resistance to 3D, 3H and 5X, but resistance to 3A was only apparent when resistance alleles were present at both loci *Rcr9^ECD01^* and *Rcr10^ECD01^* (Yu et al., 2021). It was mapped to the 12.0 to 14.5 Mb region of A08 in *B. rapa* CF v3.0, partially overlapping with *Rcr9^ECD02^* (Figure S4).

In addition, the gene *BraA.CR.b* for resistance to pathotype 3H was previously identified from the turnip differentials ECD01, ECD02, ECD03, and ECD04 and mapped to chromosome A08 (Hirani et al., 2018), but no information on the genome region corresponding to the *B. rapa* CF reference genome v3.0 was provided. Similarly, several genes for resistance to collections of *P. brassicae* from Japan and China, including *Crr1* (Suwabe et al., 2003), *CRs* (Laila et al., 2019), *PbBa8.1* (Chen, Jing, et al., 2013), and *qBrCR38‐2* (Zhu et al., 2019), have also been mapped to chromosome A08. The cloned CR gene *Crr1* was highly homologous to *Bra020861* in the *B. rapa* reference genome v1.5 and to *BraA08g014480* in the *B. rapa* reference genome v3.0, which is not located in the *Rcr9^ECD02^* genomic region. In addition, breeding lines carrying *Crr1* were not resistant to the pathotypes of *P. brassica* assessed in this study (unpublished data). Therefore, *Rcr9^ECD02^* is unlikely the same as *Crr1*. The relationship between *Rcr9^ECD02^* and *CRs* (Laila et al., 2019), *PbBa8.1* (Chen et al., 2013), and *qBrCR38‐2* needs to be determined.

The *CR* genes *CRa*, *CR^kato^*, and *Crr1* in *B. rapa* have been cloned. They all encoded TNL class disease resistance proteins (Ueno et al., 2012; Hatakeyama et al., 2013; Hatakeyama et al., 2017). There were four genes annotated as disease resistance proteins in the *Rcr9^ECD02^* interval. One of them (*BraA08g013630.3C*) belongs to the TNL class, whereas the other three (*BraA08g012910.3C*, *BraA08g012920.3C*, and *BraA08g013130.3C*) encode receptor‐like protein 30. Further studies are required to identify which of these genes is the main gene responsible for clubroot resistance, and to determine whether all four genes are necessary to show the resistance reaction to the four pathotypes. This can be addressed when the gene is cloned.

Genotyping‐by‐sequencing is a rapid, robust, inexpensive and efficient approach to identify large numbers of SNPs in a genome. In the current study, next‐generation sequencing‐based technologies were used for sequencing the parental lines and GBS of the population consisting of 93 BC_1_ plants. Whole‐genome sequencing of the parental lines ECD02 and ACDC was performed to produce high coverage of the genome (>90%) to ensure that genome‐wide polymorphic SNPs between the parental lines could be unambiguously identified. A set of high‐quality markers consisting of 1,344 SNPs distributed across the 10 chromosomes was developed and used for QTL analysis.

Similarly, KASP offers cost‐effectiveness and scalable flexibility in applications. In total, 14 robust SNP markers linked to *Rcr9^ECD02^* were developed, with six markers completely associated with the gene, and four markers flanking the gene in an interval of 2.2 cM in the population consisting of 93 lines. These SNP markers could provide an effective and robust basis for introgression of the gene into canola using marker‐assisted selection.

*Rcr9^ECD02^* on chromosome A08 of *B. rapa* for resistance to four important pathotypes (3A, 3D, 3H, and 5X) of *P. brassicae* was identified and mapped. Quantitative trait loci and major genes that were effective against pathotypes 3A, 3H, and 5X have been identified previously in other host genotypes but showed different resistance reactions to other pathotypes and were mapped to other chromosomes. *Rcr9^ECD02^* exhibited a strong and consistent resistance reaction to all four of the important pathotypes assessed, with a wider range of pathotype specificity than the other new genes for resistance, and so represents a valuable genetic resource for inclusion in new CR cultivars. Moreover, the availability of several resistance genes could provide an opportunity for the rotation of sources of resistance. Taken together, the identification of multiple clubroot resistance genes from the same general region of chromosome A08 indicates that there is a cluster of *CR* genes in the *Rcr9* interval that differs in pathotype specificity.

## AUTHOR CONTRIBUTIONS

Mizanur Rahaman: Data curation; Formal analysis; Investigation; Methodology; Validation; Writing – original draft; Writing – review & editing. Stephen E. Strelkov: Conceptualization; Supervision; Writing – review & editing. Hao Hu: Data curation; Investigation; Writing – review & editing. Bruce Gossen: Methodology; Writing – review & editing. Fengqun Yu: Conceptualization; Formal analysis; Funding acquisition; Methodology; Project administration; Supervision; Writing – original draft; Writing – review & editing.

## CONFLICT OF INTEREST

The authors declare that there is no conflict of interest.

## Supporting information

Clubroot disease severity rating scale, where: 0 = no clubbing symptoms on roots, 1 = a few small clubs, 2 = moderate clubbing, and 3 = severe clubbing on entire root.

The proportion of sequences and template coverage of 93 BC1 progeny lines of *Brassica rapa* (blue bars indicate number of sequences and orange bars indicate the percentage template coverage).

Allelic discrimination plots of KASP SNP marker analysis of ECD02 BC1 populations where ACDC is the susceptible parent and ECD02 is the resistant parent of the population.

Mapping intervals of *Rcr9*, *Rcr9^ECD02^*, *Rcr9^wa^* and *Rcr9^ECD01^* in chromosome A08 of *B. rapa* reference genome ‘Chiifu’ v3.0.

Table S1. Disease Severity Index of Brassica rapa BC1S1 lines developed from ACDC × (ACDC × T19) described by Yu et al. (2017) and B. napus controls tested with four pathotypes (3A, 3D, 3H and 5X) of Plasmodiophora brassicae.
Table S2. Number of sequences and template coverage of the whole genome sequencing of the parental lines ECD02 and ACDC, genotyping by sequencing of the 93 BC1 lines from ACDC × (ACDC × ECD02) aligned into the Brassica rapa 'Chiifu' genome v3.
Table S3. Statistics of SNP alleles from the R parent (ECD02) ‘H’ and the susceptible parent (ACDC) ‘A’ and missing data points in the BC1 derived from ACDC × (ACDC × ECD02).
Table S4. A list of SNP site in the Rcr9ECD02 target region.
Table S5. BLAST2GO search for the genes of the Rcr9ECD02 target region in the Brassica rapa ‘Chiifu’ v3.0.

## Data Availability

The data supporting this study and findings of the article are provided within the article and also with the supplementary materials. More data supporting the article would be available upon a reasonable request to the corresponding author of the article.
